# Thrombus entrapment with left atrial appendage closure to facilitate early cardioversion in tachycardiomyopathy: a case report

**DOI:** 10.1093/ehjcr/ytad618

**Published:** 2023-12-08

**Authors:** Jolie Bruno, Lorenz Räber, Bruno Schnegg, Livia Primiceri

**Affiliations:** Department of Cardiology, Bern University Hospital, Freiburgstrasse 18, 3010 Bern, Switzerland; Department of Cardiology, Bern University Hospital, Freiburgstrasse 18, 3010 Bern, Switzerland; Department of Cardiology, Bern University Hospital, Freiburgstrasse 18, 3010 Bern, Switzerland; Department of Cardiology, Bern University Hospital, Freiburgstrasse 18, 3010 Bern, Switzerland

**Keywords:** Case report, Tachycardiomyopathy, Atrial flutter, Thrombus entrapment procedure, Left atrial appendage closure

## Abstract

**Background:**

The aetiological spectrum of heart failure with reduced ejection fraction is various. Tachycardiomyopathy is recognized as one of the cause, usually made retrospectively. In this clinical context, rhythm control with restoration of sinus rhythm is considered crucial to minimize ventricular function damage and allow contractility recovery. However, the presence of a thrombus in the left atrial appendage is a limiting factor, typically requiring anticoagulation until the thrombus resolves, at least 3 weeks, thus delaying the therapy.

**Case summary:**

We present a case of 65-year-old man with diagnosis of new-onset acute symptomatic heart failure with severe reduced ejection fraction (left ventricular ejection fraction 15%), in the context of a typical tachycardic atrial flutter and concomitant thrombus in the left atrial appendage confirmed by transoesophageal echocardiography. We successfully performed a thrombus entrapment procedure by means of percutaneous left atrial appendage closure, which allowed immediate restoration of sinus rhythm through cavotricuspid isthmus ablation. After the institution of the heart failure therapy, titrated up to the maximum tolerated dose, we observed a complete restoration of left ventricular function after 6 months.

**Discussion:**

Thrombus entrapment by means of left atrial appendage closure is a valid strategy that enables early cardioversion with arrhythmia ablation and rapid restoration of normal cardiac rhythm in severe heart failure with reduced ejection fraction, even in acute situations and typical atrial flutter.

**Learning points**

- To recognize tachycardiomyopathy as a potentially reversible cause of acute heart failure.
- To consider LAA closure as a method for thrombus entrapment, enabling early cardioversion and restoration of sinus rhythm in patients with severely reduced left ventricular function caused by tachyarrhythmia.

## Introduction

Tachycardiomyopathy (TCM) is a rare but potentially reversible cause of left ventricular (LV) dysfunction. Early recognition of this entity and its treatment by eliminating the arrhythmogenic trigger are crucial to prevent haemodynamic instability and chronic irreversible changes limiting full recovery of myocardial function.^1–3^ Atrial flutter is recognized among the causes of TCM, with a prevalence of around 9% in patients referred for ablation.^4^ In view of the high success rate and the low risk of complications, ablation of the cavotricuspid isthmus (CTI) is the treatment of choice when atrial flutter and TCM are suspected.^1^ A thrombus in the left atrial appendage (LAA) represents a contraindication for any rhythm control strategy. We present a case of atrial flutter–induced TCM with LAA thrombus, undergoing percutaneous LAA closure [LAA occlusion (LAAO)] using a thrombus entrapment technique to allow immediate ablation.

## Summary figure

**Table:** 

Till May 2021	Cardiology visit: normal left ventricular ejection fraction (LVEF)
12 May 2021, 7:30 a.m.	Hospitalization for new symptomatic heart failure with reduced ejection fraction (HFrEF) in the context of tachycardic typical atrial flutter
12 May 2021, 12 p.m.	Transoesophageal echocardiography (TOE) performed to facilitate cardioversion, revealing the presence of a thrombus in the left atrial appendage (LAA)
12 May 2021, 2 p.m.	LAA occlusion procedure executed to entrap the thrombus
12 May 2021, 4 p.m.	Cardioversion achieved through cavotricuspid isthmus ablation
12 May 2021, 4:20 p.m.	Sinus rhythm restored and heart failure therapy initiated
14 May 2021	Follow-up transthoracic echocardiography (TTE) showing LVEF 35%. Progressive increase of heart failure medication
End of May 2021	Maximal tolerated doses of heart failure therapy
End of June 2021	TTE shows LVEF 45%
November 2021	Cardiology visit: normal LVEF, newly diagnosed paroxysmal atrial fibrillation
December 2021	Pulmonary vein isolation performed
December 2021 to now	No recurrence of heart failure observed

## Case presentation

A 65-year-old man presented to the emergency department complaining of palpitations and peripheral oedema, accompanied by dyspnoea on exertion for 4 weeks.

The patient had a preexisting hypertensive heart disease with reportedly normal LVEF, well-controlled blood pressure, and normal functional capacity. He had additionally a Type 2 diabetes mellitus, an ectasia of the ascending aorta, and a history of tobacco use. His undergoing treatment was perindopril/amlodipine 10/5 mg o.d., carvedilol 12.5 mg b.i.d., and metformin 1000 mg b.i.d. He was independent at home with intact cognitive function.

**Figure 1:**
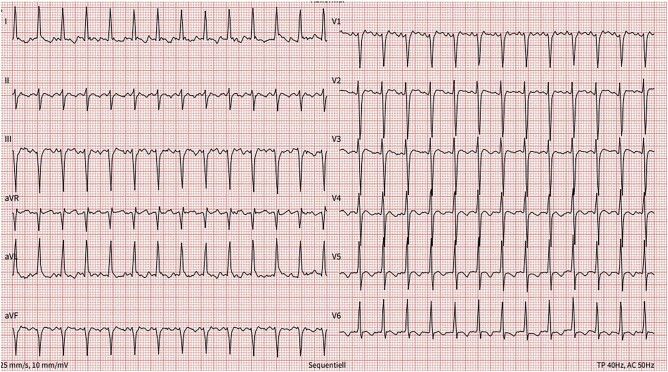
Admission electrocardiogram: typical atrial flutter, heart rate 159/min, left axis deviation, QRS 89 ms, and QTc 456 ms.

**Figure 2:**
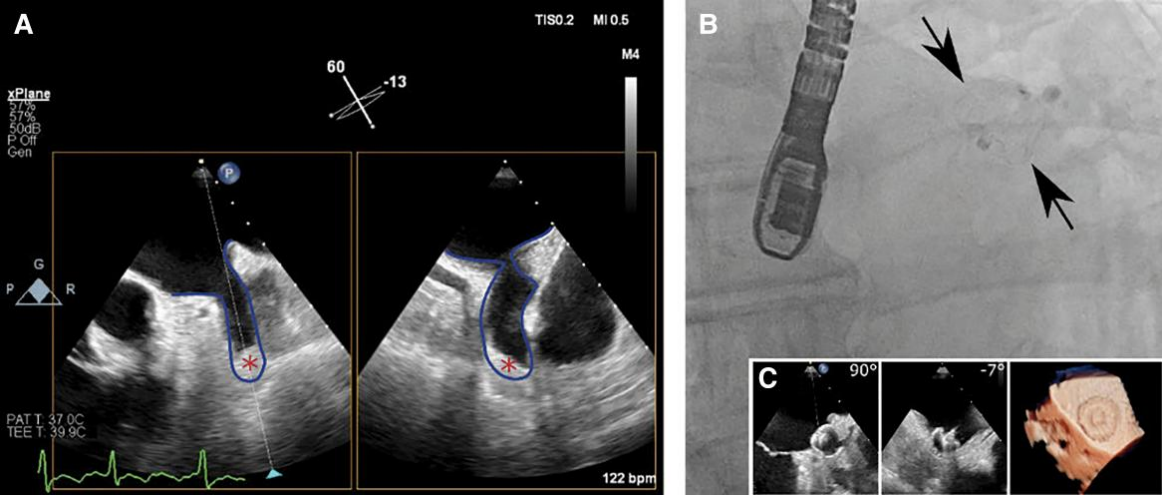
(*A*) X-plane transoesophageal view of the left atrial appendage showing a left atrial appendage thrombus. Blue line: left atrial appendage walls. Asterisk: thrombus. (*B*) Left atrial appendage closure procedure: final radiographic image of the left atrial appendage occluder placement (Watchman device). (*C*) X-plane transoesophageal view (right) and 3D view (left) showing the deployed and released Watchman device in left atrial appendage.

On physical examination, blood pressure was 144/118 mmHg, heart rate 159 b.p.m., and oxygen saturation 97% on ambient air. The auscultation revealed bibasilar crackles and regular, tachycardic heart tones. There was no jugular venous distension, but moderate peripheral oedema was noted. The electrocardiogram showed regular, narrow complex tachycardia consistent with typical atrial flutter with 2:1 conduction (*Figure 1*). Transthoracic echocardiography (TTE) revealed a newly reduced LVEF of 15% and diffuse hypokinesia, without relevant ventricular hypertrophy or dilatation (left ventricular end-diastolic diameter - 53 mm, Supplementary material online). With the aim to proceed with an electrical cardioversion (ECV), a transoesophageal echocardiography (TOE) was performed, which detected a LAA thrombus (*Figure 2A*).

The diagnosis of new acute heart failure with reduced ejection fraction (HFrEF), typical tachycardic atrial flutter, and LAA thrombus was made.

Differential diagnosis of simultaneous supraventricular tachycardia and new HFrEF includes TCM, which was highly suspected given the patient history. Laboratory tests revealed normal electrolytes and haemoglobin, serum creatinine of 110 µmol/L, and NT-proBNP 2400 pg/mL. Given the high cardiovascular risk profile, a coronary angiography was performed, which excluded a coronary artery disease.

Intravenous diuretic treatment and anticoagulation with unfractionated heparin (30,000 U/24 h for therapeutic activated partial thromboplastin time) were initiated, and the patient was transferred to the cardiac care unit for further management. Restoration of sinus rhythm was considered essential to allow contractility recovery. However, ECV and catheter ablation were contraindicated because of the LAA thrombus.

**Figure 3:**
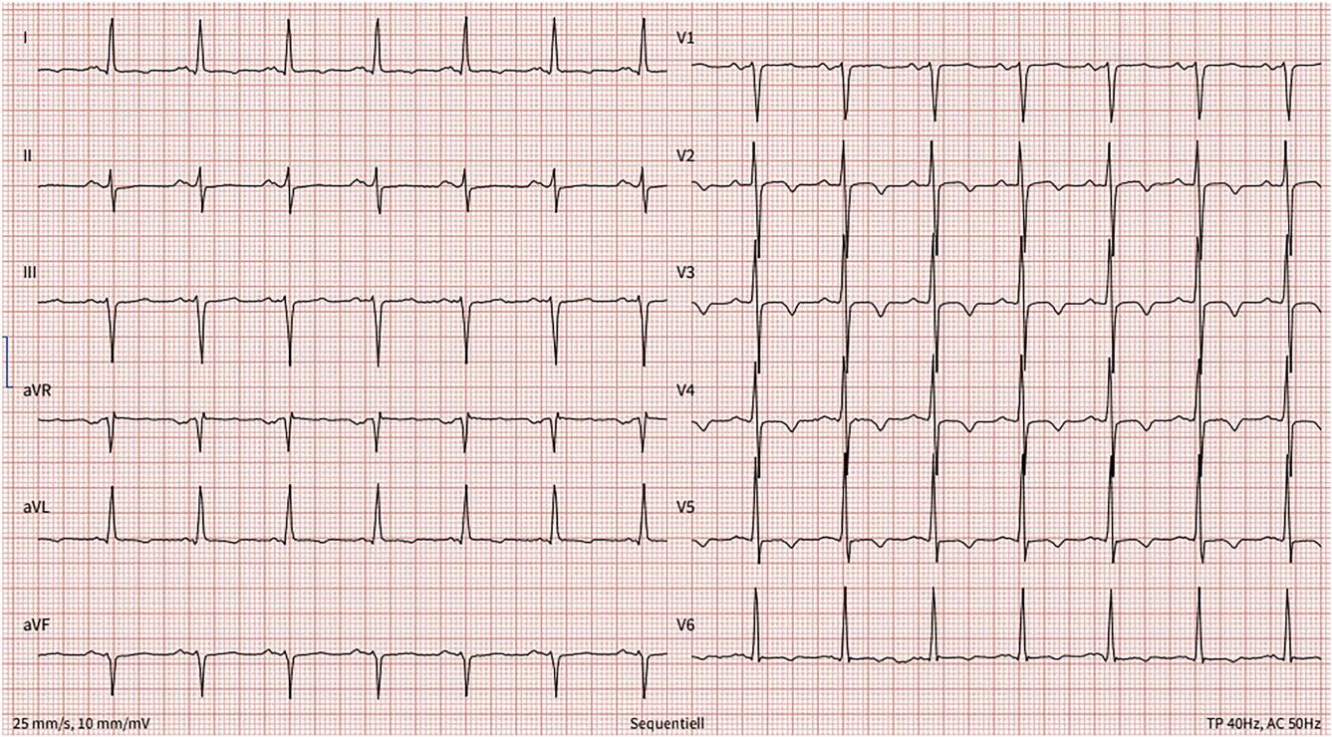
Discharge electrocardiogram: normocardic sinus rhythm, heart rate 91/min, left axis deviation, PQ 160 ms, QRS 90 ms, negative T-waves in I, aVL, V2–6, and QTc 444 ms.

We decided to perform a percutaneous LAAO using a thrombus-trapping technique with a 20 mm Watchman FLX (*Figure 2B* and *C*). Under sedo-analgesia and TOE guidance, a Watchman 14 F delivery sheath was placed in the upper left pulmonary vein following transseptal puncture at an infero-posterior localization. The Watchman device was advanced through the sheath and partially expanded inside the pulmonary vein (so-called ball configuration). With the atraumatic ball configuration, the sheath was directed towards the anticipated landing zone in the LAA neck and finally released by unsheating the catheter. After TOE verification of device compression and exclusion of residual leak, the device was released. On the same day and separate intervention, a successful ablation of the CTI was performed with restoration of sinus rhythm (*Figure 3*).

The therapy with heparin was changed to apixaban 5 mg b.i.d. for 3 months (after ablation) in combination with clopidogrel 75 mg o.d. for 12 months. Heart failure therapy was started including metoprolol 25 mg b.i.d., spironolactone 25 mg o.d., and dapagliflozin 10 mg o.d. and a switch of candesartan 8 mg b.i.d. to sacubitril/valsartan 25 mg b.i.d. till 75 mg b.i.d. at discharge. In the TTE following the two procedures, a device embolization and pericardial effusion were excluded, and an improvement of the LV function to 35% was already noted (see Supplementary material online). Forty-five days after discharge, a TOE attested a LVEF of 45% and absence of device-related thrombosis or any peri-device leaks. The 6-month TTE (see Supplementary material online) revealed a normalized LV function under maximally tolerated doses of heart failure therapy including sacubitril/valsartan 200 mg b.i.d., spironolactone 25 mg o.d., dapagliflozin 10 mg o.d., and metoprolol 25 mg b.i.d. The patient was asymptomatic and feeling well. A 7-day ECG confirmed the absence of recurrence of atrial flutter but identified episodes of paroxysmal atrial fibrillation, leading to pulmonary vein isolation. No thromboembolic events or recurrence of heart failure occurred under the prescribed therapy, which was unchanged in the course.

## Discussion

TCM is a ventricular dysfunction in response to rapid and/or asynchronous or irregular myocardial contraction, partially or completely reversible after treatment of the arrhythmic trigger and not instantaneous.^1–3^ The diagnosis may only be confirmed *ex juvantibus*.

An early diagnosis of TCM is valuable because of the potential for recovery with appropriate treatment, specifically treating the proarrhythmic trigger.^1–3^

The presence of a LAA thrombus excluded the possibility to perform a direct ECV. Given the less concealed conduction into the atrioventricular node (and therefore more difficult rate control) of the atrial flutter, a rate control strategy has often limited success.^1^

The safety and efficacy of percutaneous LAAO using a thrombus entrapment technique were previously described in cases without acute presentation.^5^ In our case, this procedure allowed immediate ECV by ablation of atrial flutter. The potential risk of percutaneous LAAO using the thrombus entrapment technique is thrombus embolization. Several criteria should be fulfilled to allow a safe procedure. First, there should be sufficiently large device landing zone free of thrombus, with anticipated co-axial device delivery. This enables a device implantation without recapture, a step that would ultimately increase the risk for thrombus embolization. Second, the thrombus should be located in the depth of the LAA. Third, the device size needs to be precisely assessed by TOE, ideally based on multiplanar assessment of the landing zone, to avoid device recaptures due to erroneous sizing. For the procedure, it is relevant not to insert/manipulate catheters into the depth of the LAA and not to perform LAA angiography. In variance to a standard LAAO procedure, the catheter sheath is not placed inside the LAA but rather in the upper left pulmonary vein and directed towards the LAA ostium, with a partially released device (ball configuration) that provides an atraumatic tip, avoiding LAA injury and tamponade. Regarding device selection, the Watchman FLX appears ideal as it has more self-adapting capability if the delivery sheath position is not entirely co-axial to the landing zone. Furthermore, patient requiring oral anticoagulation following LAAO (in this case due to ablation procedure) might have a lower risk of late-onset pericardial effusion with the Watchman as compared to the Amulet device.^6^

Antithrombotic therapy post-intervention is important to prevent device-related thrombus during the endothelialization. Depending on the risk of bleeding, different regimens have been proposed.^7^ For this procedure, the optimal strategy has to be determined,^5^ particularly if antiplatelet therapy is needed on top of anticoagulation that is mandatory after ablation procedure.

The limitation of this strategy is the availability of the procedure and that is still off-label, although reportedly safe.^5^

Thrombus entrapment by means of percutaneous LAA closure is a valid treatment option for patients with TCM with contraindication for ECV due to LAA thrombus.

## Supplementary Material

ytad618_Supplementary_Data

## Data Availability

All data are incorporated into the article and its online Supplementary material.

## Lead author biography

Jolie Bruno, MD, is a resident physician in cardiology at Bern University Hospital, Inselspital, in Switzerland. Her primary interests lie in the field of heart failure, acute cardiovascular care, and interventional cardiology, where she intends to pursue further studies, practice, and research.
